# The orchid seed coat: a developmental and functional perspective

**DOI:** 10.1186/s40529-023-00400-0

**Published:** 2023-09-27

**Authors:** Yung-I. Lee, Edward C. Yeung

**Affiliations:** 1 Department of Life Science, National Taiwan University, Taipei, 10617 Taiwan; 2 Institute of Ecology and Evolutionary Biology, National Taiwan University, Taipei, 10617 Taiwan; 3 Department of Biological Sciences, University of Calgary, Calgary, AB T2N 1N4 Canada

**Keywords:** Inner and outer integument, Unitegmic integument, Bitegmic integument, Seed coat, Carapace, Embryo, Funiculus, Micropyle

## Abstract

Orchid seeds are 'dust-like.' The seed coat is usually thin, with only one to a few cell layers. It originates from the integuments formed during ovule development. In orchids, the outer integument is primarily responsible for forming a mature seed coat. The inner integument usually fails to develop after fertilization, becomes compressed, and collapses over the expanding embryo. Hence, the seed coat is formed from the funiculus, chalaza, and outer integumentary cells. The outermost layer of the seed coat, the testa, is lignified, usually at the radial and inner tangential walls. The subepidermal thin-walled layer(s), the tegmen, subsequently cold, resulting in seeds having only a single layer of seed coat cells. In some species, cells of the inner integument remain alive with the ability to synthesize and accumulate lipidic and or phenolic compounds in their walls covering the embryo. This cover is called the 'carapace,' a protective shield contributing to the embryo's added protection. A developmental and functional perspective of the integuments and seed coat during seed development and germination is presented in this review.

**Supplementary Information:**

The online version contains supplementary material available at 10.1186/s40529-023-00400-0.

## Background

In seed plants, the embryo is protected by a seed coat originating from the integument(s) formed during ovule development. The importance of the seed coat in seed development and germination is well recognized and a subject of reviews (Mohamed-Yasseen et al. 1994; Boesewinkel and Bouman 1995; Moise et al. 2005; Radchuk and Bonisjuk 2014; Matilla 2019). Since the seed coat completely encloses the embryo, the essential functions are to supply nutrients to the developing embryo and offer physical protection during embryo development and germination. In past decades, as the published literature shows, additional information on seed coat formation and function has been elaborated, e.g., as in Brassicaceae species (Raviv et al. 2017) and tomato (Chaban et al. 2022), indicating the uniqueness and importance of the seed coat. The evolution and molecular control of seed coat development have also been summarized recently by Matilla (2019).

Orchid seeds are 'dust-like.' The seed coat is usually thin. The inner seed coat, the tegmen, tends to collapse as the seed matures, leaving an outer layer, the testa, with distinct surface features. Comprehensive information on orchid seed morphology and seed coat structure is available, i.e., Dressler (1993), Rasmussen (1995), and Molvray and Chase (1999). Arditti and Ghani (2000) detailed the numerical and physical characteristics of orchid seeds in an extensive review. Barthlott et al. (2014) provided a scanning electron microscopy survey of orchid seed diversity, illustrating the seed coat's surface features and morphology.

In the study of orchid seeds, most investigations focus on surface features relating seed morphology to seed dispersal and biosystematics discussion, e.g., Molvray and Kores (1995), Gamarra et al. (2007), Hariyanto et al. (2020), and Aprilianti et al. (2021). Collier et al. (2023) recently reported differences in seed morphometrics of orchids native to North America and Hawaii. Their goal is "a better understanding of seed morphometrics, and especially the structure and function of the testa may be useful in developing more effective protocols aimed at in vitro seed germination." Moreover, detailed ontogenetic accounts of seed coat formation in orchids are not readily available in the literature. Furthermore, its potential functions during embryo development are seldom discussed. Recently, Yeung (2022) reported that the inner integument in *Epidendrum ibaguense* takes on an active cytological appearance with wall ingrowths at fertilization and proembryo development. This observation draws attention to the integuments in seed formation and warrants further investigation.

**Fig. 1:**
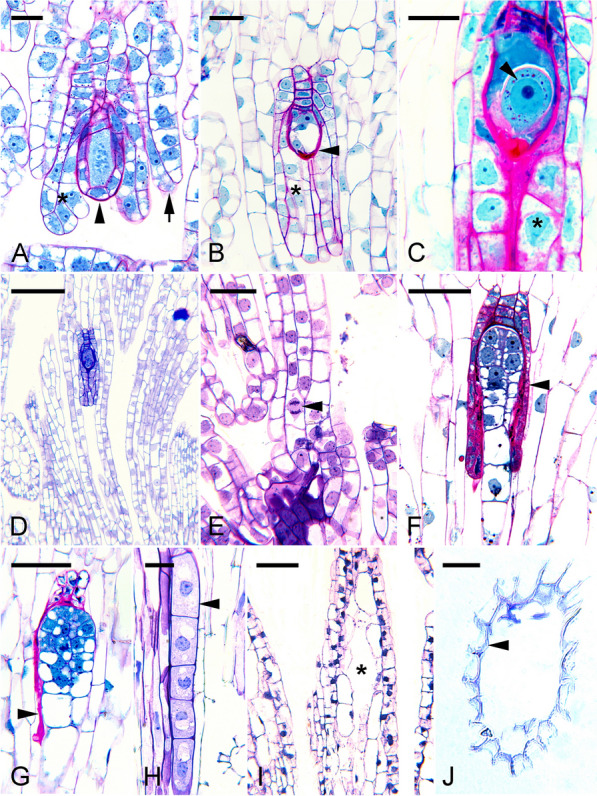
HYPERLINK "sps:id::fig1||locator::gr1||MediaObject::0"The ovule and seed development of *Epidendrum ibaguense*. **A** The archesporial cell enlarges and differentiates into the megasporocyte and is enveloped by a single layer of nucellar cells (arrowhead). At the same time, both the inner (*) and outer (arrow) integuments have developed. Scale bar = 20 μm. **B** A mature embryo sac (arrowhead) showing the egg apparatus. The inner integument (*) is well developed at the micropylar end, forming the micropyle. The outer integument has extended beyond the inner integument as the ovule matures. Scale bar = 50 μm. **C** After fertilization, the zygote (arrowhead) has a dense cytoplasm with a prominent nucleus and some starch deposits (small red dots). The inner integumentary cells (*) at the micropylar end become densely cytoplasmic; each cell has a distinct nucleus. The walls of the inner integumentary cell thicken, and wall ingrowths are present. Scale bar = 20 μm. **D** A lower magnification micrograph giving a general overview of the contrasting staining intensity between the inner and outer integuments. The fertilized ovule and the inner integument have a stronger staining intensity compared to the vacuolated outer integumentary cells. Scale bar = 150 μm. **E** A narrow funiculus connects the developing seed to the maternal placental tissue. Mitotic activity (arrowhead) can be discerned at the time of fertilization. Scale bar = 40 μm. **F** As the proembryo increases in size and the suspensor begins to protrude beyond the opening of the inner seed coat, the cells of the inner seed coat (arrowhead) gradually become compressed. Scale bar = 50 μm. **G** The embryo continues to increase in size. As a result, the inner seed coat is crushed, and only remnants (arrowhead) remain adhering to the embryo proper. Scale bar = 50 μm. **H** Light micrograph showing a portion of the suspensor (arrowhead) pressing against the walls of the seed coat cells. The inner layers of the seed coat stain purple with the TBO stain, indicating the absence of phenolic compounds in the walls. Scale bar = 10 μm. **I** Fewer mitotic divisions in the inner chalazal cells result in creating a cavity (*) during seed development. Scale bar = 60 μm. **J** Light micrograph showing a TBO-stained section of a mature seed coat (arrowhead). Judging from the staining reaction, there is a preferential deposition of lignin in the seed coat's inner periclinal and radial walls. Scale bar = 50 μm

**Fig. 2:**
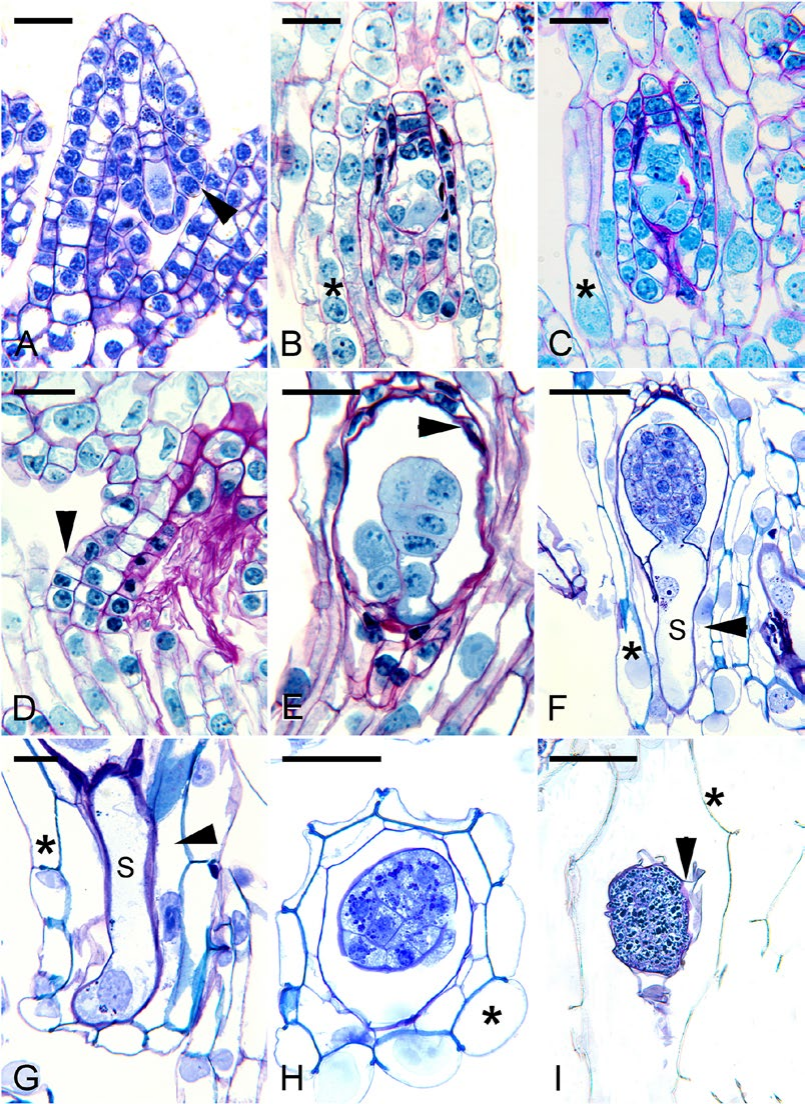
The ovule and seed development of *Phaius tankervilliae*. **A** Light micrograph of a megasporocyte. Both integuments (arrowhead) have just been initiated and become visible. Scale bar = 40 μm. **B** Light micrograph of a mature embryo sac. The inner integument has enclosed the embryo sac and results in the formation of a micropyle (arrowhead). The cells of the outer integument (*) have extended beyond the micropyle. Scale bar = 80 μm. **C** At fertilization, the pollen tube has penetrated the embryo sac through the micropyle (arrowhead). The cells of the outer integument (*) elongate further and become highly vacuolated. Scale bar = 80 μm. **D** The developing seed attaches to the placenta through the funiculus (arrowhead). The cells of the funiculus at the junction are small in size and will elongate as the seed develops. Scale bar = 50 μm. **E** Light micrograph showing a proembryo and a degenerated endosperm nucleus next to the zygote. At this stage, the inner seed coat (arrowhead) is collapsing. Scale bar = 60 μm. **F** A longitudinal section showing the embryo at the globular stage with an elongated suspensor (S). Scale bar = 80 μm. **G** The suspensor cell (S) elongates and tightly presses against the cell lining of the seed coat. Secondary walls begin to form in the outermost seed coat layer (*), while cells of the inner layer (arrowhead) remain cytoplasmic with a prominent nucleus (arrowhead). Scale bar = 25 μm. **H** A cross-section showing a globular embryo enclosed by the seed coat. The seed coat is composed of two cell layers. Lignin has deposited in the inner tangential and anticlinal walls of the outer cell layer (*). The inner layer will subsequently collapse and fuse with the outer cell layer. Scale bar = 100 μm. **I** At maturity, the seed coat (*) comprises a single layer of cells with lignified inner tangential and radial walls. Remnants of the collapsed wall from the inner integument (arrowhead) can be found outside the embryo. Scale bar = 80 μm

**Fig. 3:**
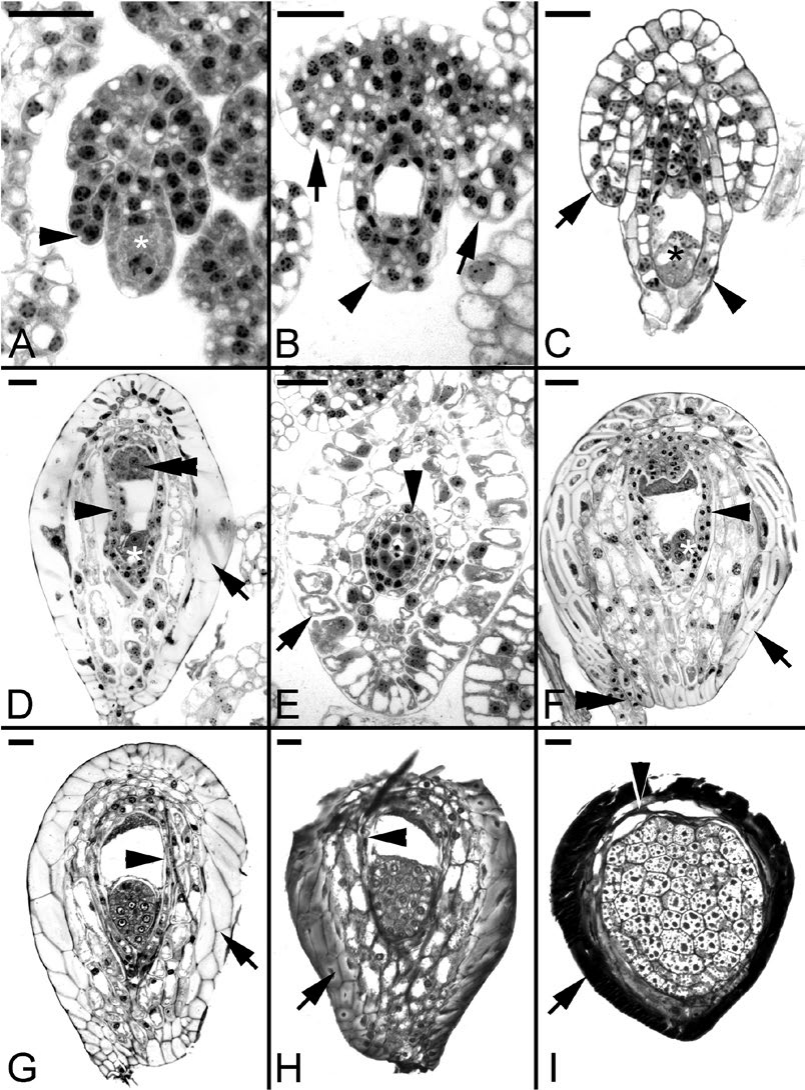
The ovule and seed development of *Vanilla planifolia*. **A** The archesporial cell enlarges and differentiates into the megasporocyte (*). At the same time, the inner integument (arrowhead) has become visible. Scale bar = 30 μm. **B** Light micrograph showing an expanding four-nucleate embryo sac by vacuolation. The inner integument (arrowhead) has completely enclosed the embryo sac, and the multilayered outer integument (arrows) is expanding. Scale bar = 30 μm. (C) A mature embryo sac showing the egg apparatus (*), including the egg cell and two synergids. The outer integument (arrow) continues to elongate; moreover, it has not enclosed the inner integument (arrowhead). Scale bar = 30 μm. **D** After fertilization, the zygote (*) has a dense cytoplasm, and the degenerated antipodal cells (double arrowhead) at the chalazal end are also densely stained. At this stage, the cytoplasm of the inner seed coat (arrowhead) remains densely stained, and the walls of the outermost layer of the outer seed coat (arrow) become thickened. Scale bar = 30 μm. **E** Light micrograph showing a cross-section of a fertilized ovule showing the distinct layers of the inner seed coat (arrowhead) and outer seed coat (arrow). Scale bar = 30 μm. **F** Light micrograph showing a three-celled proembryo (*), and the developing seed attaches to the placenta through the funiculus (double arrowhead). The inner seed coat (arrowhead); The outer seed coat (arrow). Scale bar = 30 μm. **G** Light micrograph showing an early globular embryo without a distinct suspensor during embryo development. At this stage, the inner seed coat (arrowhead) gradually compresses. The outer seed coat (arrow). Scale bar = 30 μm. **H** Light micrograph showing a globular embryo. At this stage, the inner seed coat (arrowhead) has shriveled, and the outermost layer of the outer seed coat (arrow) has become lignified. Scale bar = 30 μm. **I** At maturity, numerous tiny protein bodies are found within the embryo proper cells. In this preparation, the lipid bodies are not preserved; storage lipid bodies occupy the spaces between the protein bodies. The inner seed coat (arrowhead) has compressed into a thin layer, and the outermost layer of the outer seed coat (arrow) has lignified and been filled with dark material. Scale bar = 30 μm

**Fig. 4:**
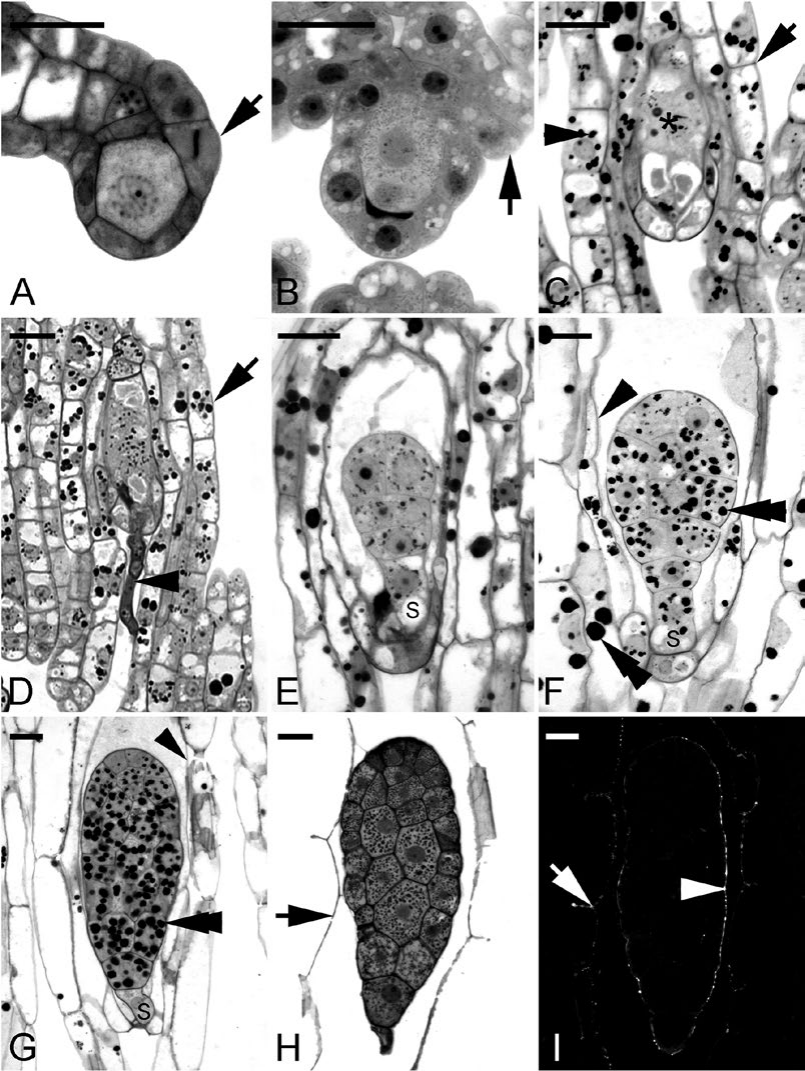
The ovule and seed development of Gastrodia nantoensis. **A** The archesporial cell is differentiating into a megasporocyte. Cell division (arrow) near the ovule's chalazal end signifies the integument tissue's initiation. Scale bar = 20 μm. **B** The second meiotic division results in the formation of two megaspores of unequal size. At the same time, the initiation of integument tissue is becoming visible (arrow). Scale bar = 20 μm. **C** A longitudinal section through a mature embryo sac showing the egg apparatus (*). The integument tissue (arrow) has completely enclosed the embryo sac at this stage. Starch grains (arrowhead) start to accumulate in the integument tissue. Scale bar = 20 μm. **D** At the time of fertilization, the pollen tube (arrowhead) penetrates the embryo sac, and the integument tissue elongates further and becomes the seed coat (arrow). Scale bar = 20 μm. **E** Light micrograph showing a proembryo with a suspensor cell (S). Scale bar = 20 μm. **F** A longitudinal section through a developing globular embryo. At this stage, the nucellus (arrowhead) gradually compresses, and large starch grains (double arrowheads) are abundant in the cells of the embryo proper and the seed coat. The suspensor cell (S). Scale bar = 20 μm. **G** As the seed approaches maturity, starch grains (double arrowhead) are prominent within the embryo cells, and the suspensor cell (S) has reduced its size and begins to degenerate. At this stage, the nucellus (arrowhead) has compressed and degenerated. Scale bar = 20 μm. **H** At maturity, the embryo has smaller cells near the chalazal end and larger cells in the micropylar end. The suspensor has degenerated at this stage, and the embryo proper is enveloped by a shriveled seed coat (arrow). Scale bar = 20 μm. **I** Nile red staining fluorescence micrograph of a mature seed at the same stage as that seen in Fig. 4I. The seed coat (arrow) and the surface wall (arrowhead) of the embryo proper react positively to the stain. Scale bar = 20 μm

**Fig. 5:**
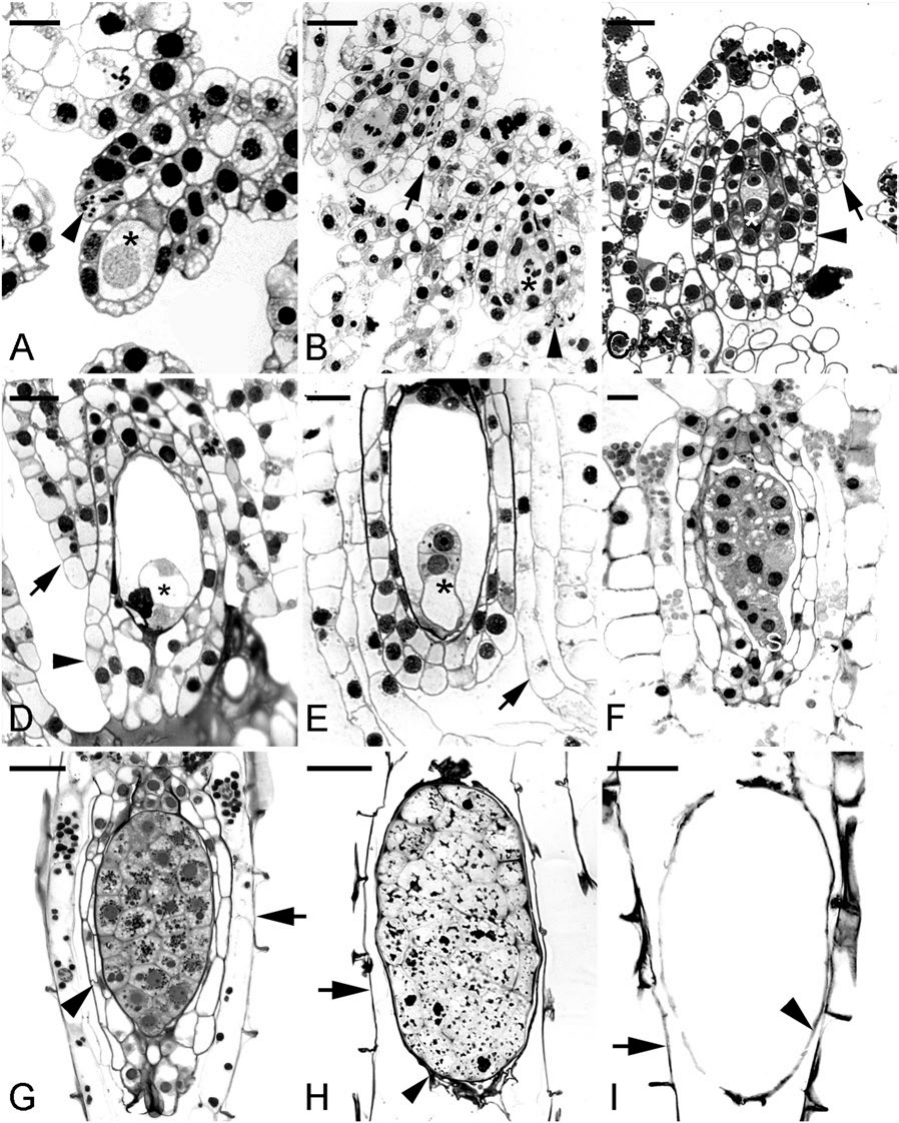
The seed coat development of *Cypripedium formosanum*. **A** The archesporial cell (*) differentiates into a megasporocyte. Cell division (arrowhead) near the ovule's chalazal end signifies the inner integument's initiation. Scale bar = 20 μm. **B** Light micrograph showing the first meiotic division at the metaphase (*). At this stage, the inner integument (arrowhead) is expanding but has not enclosed the megasporocyte completely, and the outer integument has initiated (arrow). Scale bar = 40 μm. **C** The second meiotic division forms a two-nucleate embryo sac (*). At this stage, the inner integument (arrowhead) has enclosed the developing embryo sac completely, and the outer integument is still growing (arrow). Scale bar = 40 μm. **D** Light micrograph of a zygote (*) just after fertilization. The zygote is polarized with a chalazal-located nucleus and a prominent vacuole occupying the micropylar end. The embryo sac and the inner seed coat (arrowhead) have enlarged and elongated at this stage. In contrast, the expanding outer seed coat (arrow) has not enveloped the entire embryo sac. Scale bar = 30 μm. **E** Light micrograph of a two-celled embryo (*), and the cell division is unequal as judged from the location of the newly formed cell plate. The outer seed coat (arrow) envelopes the embryo sac at this stage. Scale bar = 30 μm. **F** Light micrograph of an early globular embryo. This species has a single-celled suspensor (S) that does not protrude from the embryo sac throughout embryo development. Scale bar = 30 μm. **G** Light micrograph showing a maturing globular embryo. At this stage, storage products, such as protein bodies, accumulate within the cells of the embryo proper. The cells of the inner seed coat (arrowhead) begin to shrivel, and the out layer of the outer seed coat (arrow) has compressed. Scale bar = 40 μm. **H** At maturity, the embryo proper is enveloped by the shriveled inner seed coat (arrowhead) and outer seed coat (arrow). Scale bar = 40 μm. **I** Light micrograph showing a longitudinal section through the inner seed coat (carapace; arrowhead) and outer seed coat (arrow) with the embryo proper removed. Scale bar = 40 μm

**Fig. 6:**
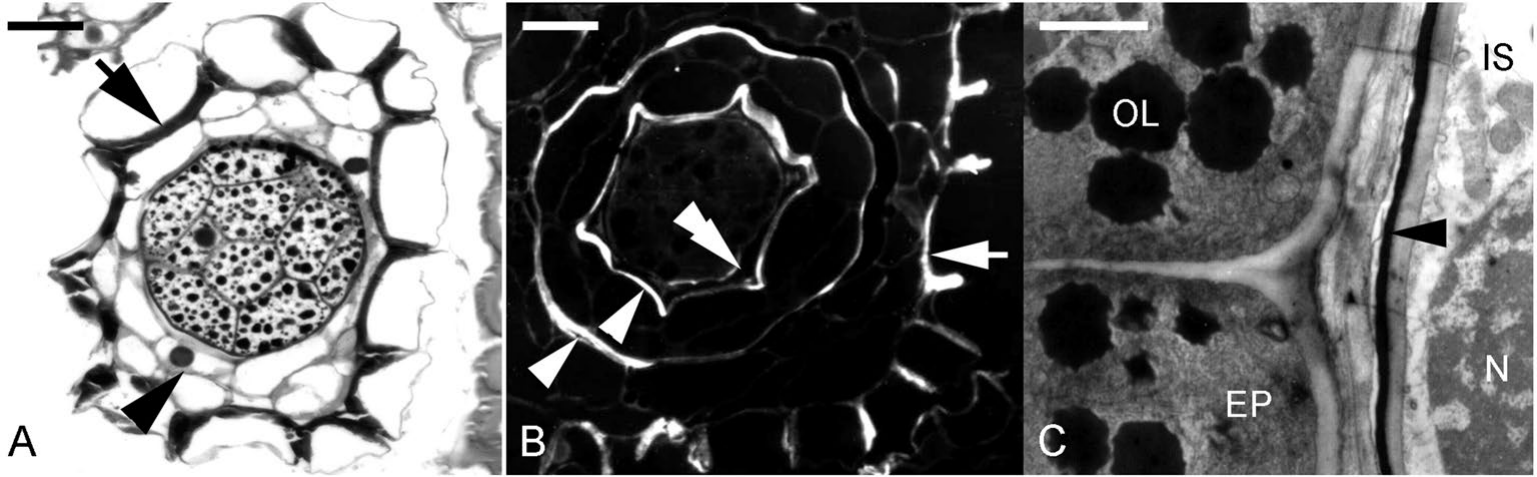
The formation of carapace in *Cypripedium formosanum* seeds. **A** Light micrograph showing a cross-section through a developing seed at the globular stage. The embryo is enclosed by the inner seed coat (arrowhead) from the inner integument. The tangential and radial walls of the outer seed coat (arrow) have thickened. Scale bar = 30 μm. **B** Light micrograph showing the fluorescence pattern of a cross-section through a developing seed at the stage similar to **A** after the Nile red staining. The surface wall of the embryo proper possesses fluorescent signals (double arrowheads). In addition, the tangential and radial walls of the outer seed coat (arrow) and the inner and outer surface walls of the inner seed coat (arrowheads) fluoresce brightly. Scale bar = 30 μm. **C** Electron micrograph showing the adjoining region of the embryo proper cell and the cell of the inner seed coat at the globular stage. Osmiophilic lipid bodies (OL) have accumulated within the embryo proper cell (EP), and a distinct osmiophilic layer (arrowhead) is present in the inner surface wall of the inner seed coat (IS). N, nucleus. Scale bar = 2 μm

**Fig. 7:**
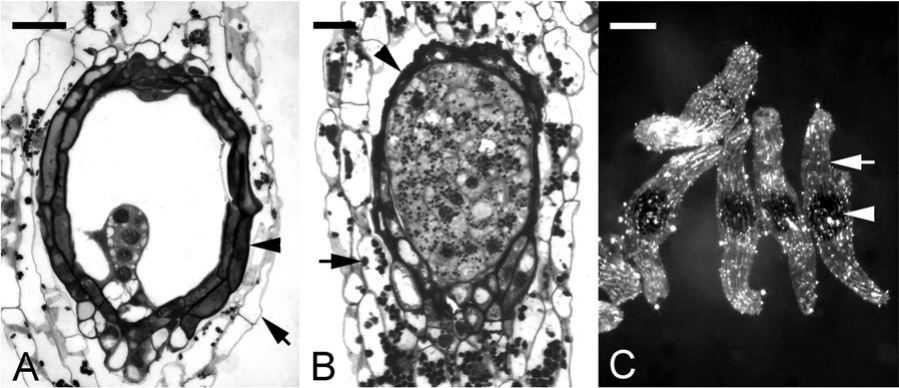
The formation of carapace in *Cypripedium plectrochilum* seeds. **A** At the proembryo stage, the cell wall of the inner seed coat (arrowhead) has become thickened, and the cytoplasm of the inner seed coat is filled with dark materials. The cell wall of the outer seed coat (arrow) remains thin-walled without lignification, as judged by the purple color of the TBO stain. Scale bar = 30 μm. **B** Light micrograph showing maturing globular embryo. At this stage, the cells of the inner seed coat begin to shrivel, resulting in the formation of a black and thickened carapace. At the same time, the outer seed coat is still alive with starch deposits within the cytoplasm. Scale bar = 30 μm. **C** In the mature seeds of *C. plectrochilum*, the embryo is enclosed by a black and thickened inner seed coat, i.e., carapace (arrowhead), and then enveloped by the thin-walled outer seed coat (arrow). Scale bar = 200 μm

**Fig. 8:**
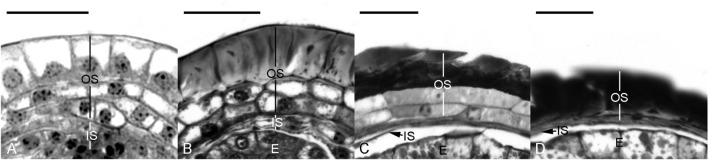
The seed coat development of *Vanilla planifolia*. **A** The seed coat consists of an inner seed coat (IS, two cells thick) and an outer seed coat (OS, three to four cells thick). At the time of fertilization, the cell wall of the outermost layer of the outer seed coat remained primary in nature. Scale bar = 20 μm. **B** In the globular embryo stage, the cell wall of the outermost layer of the outer seed coat (OS) thickens, and the inner seed coat (IS) becomes dehydrated and compressed. Scale bar = 20 μm. **C** As the seed matures, the thickened outermost layer of the outer seed coat and the inner layers gradually dehydrate and compress. The inner seed coat (IS) has compressed into a thin layer at this stage. Scale bar = 20 μm. **D** At maturity, both the thin inner seed coat (IS) and the thickened outer seed coat (OS) compress and envelop the embryo **E** tightly. Scale bar = 20 μm

The primary objective of this review is to illustrate the structural features and different patterns of seed coat formation in selected orchid species, as shown in Figs. 1, 2, 3, 4, 5, 6, 7, 8 and to discuss seed coat functions during seed development and germination. Questions and suggestions are included in the discussion, hoping to generate more interest and debate in studying the orchid seed coat. Embryo development in orchids is unusual, and many questions remain, especially on regulating its development (Yeung 2022). Understanding the orchid seed coat can provide additional insights into the embryo and seed development.

## Integument formation during orchid ovule development

The integuments become the seed coat after fertilization. In most orchid species, integuments form during megasporogenesis (Yeung and Law 1997). The inner integument usually initiates earlier than the outer integument. The bitegmic condition of ovules, i.e., having two integuments, is most common in orchids. Moreover, variations in integument formation and structural organization are noted. Orchid ovules having a single integument are also known, e.g., *Epipogium aphyllum* (Kusano 1915; Afzelius 1954), *Gastrodia elata* (Abe 1976; Li et al. 2016), and *Paphiopedilum godefroyae* (Ren and Wang 1987). The single integument is highly reduced in size in *Epipogium roseum* (Arekal and Karanth 1981), and it does not cover the nucellus of a mature embryo sac and lacks a distinct micropyle (Additional file 1). And recently, a species, *Pogoniopsis schenckii*, with ategmic ovules, have been reported (Alves et al. 2019); only the nucellus encloses the embryo sac and subsequently becomes the seed coat. Abe (1972) considered species with unitegmic ovules to be more advanced from an evolutionary perspective.

### Integument formation in orchids with bitegmic ovules

#### *Epidendrum ibaguense*—a tropical epiphyte orchid

In *E. ibaguense*, the integuments initiate during the archesporial cell formation (Yeung and Law 1989; Yeung 2022). Surface nucellar cells begin to divide near the archesporial cell of the ovular primordia. The inner integument develops rapidly, enclosing the megasporocyte (Fig. 1A). It is two cells thick. Moreover, the integumentary cells at the micropylar end of the ovule have additional divisions, forming a prominent micropyle (Fig. 1B–D). The inner integumentary cells increase in cytoplasmic density, especially during fertilization and proembryo development (see Fig. 1C in Yeung 2022).

The outer integument initiates later than the inner integument and develops near the chalaza. Periclinal walls in the nucellar epidermis mark its initiation (Yeung and Law 1989). When the mature embryo sac forms, the outer integument has overtaken the inner integument. It is a bilayer structure and continues to elongate and extends toward the funiculus (Fig. 1D).

The *E. ibaguense* ovule takes on an anatropous orientation, with the micropyle facing the placental tissue. The funiculus connecting the ovule to the placenta is narrow and approximately four cells thick. At the funiculus-placenta junction, cells are small and mitotic figures are visible (Fig. 1E). After fertilization, cells at the junction elongate to accommodate seed elongation. By the time of fertilization, the single-layered nucellar tissue is crushed by the expanding embryo sac and is difficult to discern.

#### *Phaius tankervilliae*—a subtropical terrestrial orchid

*P. tankervilliae* (Fig. 2), commonly known as "the Nun orchid"—a subtropical terrestrial orchid, its integument formation resembles *E. ibaguense* with the initiation of the inner integument at the time of megasporocyte formation (Fig. 2A). This is soon followed by the appearance of the outer integument. The inner integument consists of a single cell layer except at the micropylar end, where it becomes a bilayer (Fig. 2B, C). The outer integument extends beyond the inner integument as the ovule matures (Fig. 2B). As in *E. ibaguense*, the cells of the inner integument at the micropyle enlarge with increased cytoplasmic density at the time of fertilization (Fig. 2C). The nucellar tissue surrounding the mature embryo sac is compressed and becomes difficult to discern. The funicular cells connecting the placental tissues remain small at fertilization and will elongate to accommodate seed growth after fertilization (Fig. 2D).

#### *Calypso bulbosa*—a temperate terrestrial orchid

In *C. bulbosa*, a temperate terrestrial orchid, the inner integument initiates near the archesporial cells during ovule development (for micrographs, see Law and Yeung 1989; Yeung and Law 1992). It develops rapidly until it completely encloses the tip of the nucellus containing the developing megaspores. The outer integument grows slowly and does not extend beyond the inner integument before fertilization. Thus, the micropyle is formed from the inner integument. Elongation of the outer integument takes place after fertilization. In *C. bulbosa*, starch granules are present in the outer integument and cells of the chalazal tissues, especially after fertilization. Similar to *E. ibaguense*, the cells of the inner integument increase in cytoplasmic density during fertilization and proembryo development (Yeung and Law 1992).

### *Vanilla* species as examples of bitegmic ovules with multicell-layered integuments

*Vanilla*, a tropical orchid genus, is known for having a thick and hard seed coat. The seedling growth is initially terrestrial. As the vine continues to grow and climbs up trees, the orchid becomes epiphytic. Because of its economic importance, information about its reproductive biology is readily available in the literature, e.g., Swamy (1947), Nishimura and Yukawa (2010), Kodahl et al. (2015), and Yeh et al. (2021). The thick seed coat originates from multilayered integuments of the ovule before fertilization. In *V. planifolia*, the inner integument differentiates during archesporial cell formation and is composed of two to three cell layers (Fig. 3A). It envelops the developing ovule before the completion of megasporogenesis. The outer integument appears when the megasporocyte undergoes meiotic divisions. It comprises three to four cell layers with additional layers at the chalazal end (Swamy 1947) (Fig. 3B). In *V. imperialis* (Kodahl et al. 2015), integument formation is similar to *V. planifolia*, except for differences in the timing of integument initiation and nucellar cell degeneration.

Unlike the inner integument, the multilayer outer integument grows slowly and does not envelop the inner integument before fertilization (Fig. 3C). Moreover, at fertilization, the outer integument grows rapidly and completely encloses the embryo sac and the inner integument (Fig. 3D). Notably, the cells of the outermost layer of the outer seed coat enlarge rapidly during fertilization; the outer tangential and radial walls become thickened considerably (Fig. 3D). The thickened wall stained pinkish-red with the toluidine blue O (TBO) stain indicates the thickened wall remains primary in character.

### *Gastrodia* and* Epipogium* species as examples of orchids with unitegmic ovules

Some mycoheterotrophic orchids, such as *Gastrodia* and *Epipogium*, have ovules with a single integument, termed the unitegmic ovule (Tohda 1967; Abe 1976; Arekal and Karanth 1981; Li et al. 2016). In *Gastrodia* species, i.e., *G. elata* and G. *nantoensis*, a single layer of nucellar cells encloses the developing megaspores and, subsequently, the embryo sac (Li et al. 2016). A single integument initiates during megaspore formation (Fig. 4A, B). As the ovule matures, the integumentary cells elongate rapidly, eventually enclosing the embryo sac, leaving a micropyle opening (Fig. 4C). Prominent starch granules are present in the integumentary and chalaza tissues during ovule development (Fig. 4C). Similar to G. *nantoensis*, the single integument of *E. roseum* has not covered the nucellus of a mature embryo sac at the time of fertilization (Additional file 1). The integument tissue encloses the fertilized embryo sac after the first cell division of the zygote and becomes the seed coat. Although the unitegmic ovule has simpler integument structures, the pollen tube's guidance and the synergids' penetration still occur normally in the absence of a distinct micropyle (Fig. 4D).

## The roles of the integuments

The integuments formed during ovule formation are programmed to become the seed coat after fertilization. Moreover, judging from its developmental patterns and cytological features, the inner integument appears to take on functional roles during the ovule and early embryo development. Whereas the outer integument functions in seed coat formation after fertilization. The fact that outer integument is not necessarily developed at fertilization, as shown in *Cremastra appendiculata* (Abe 1968) and *Calypso bulbosa* (Law and Yeung 1989), and it does not take part in micropyle formation such as *Bletilla striata* (Abe 1971), indicates that it is programmed to function in seed coat formation after fertilization.

Although there are indications that the inner integument possesses unique biochemical properties, its importance in development tends to be overlooked. A high peroxidase activity has been localized histochemically in the inner integument of *Encyclia tampensis* (Alvarez 1968) and the micropylar region of the integument in *Cypripedium* (Zinger and Poddubnaya-Arnoldi 1966). A marked activity of dehydrogenases has also been detected in the ovules' integument in several orchids (Zinger and Poddubnaya-Arnoldi 1966). These earlier studies indicate that the inner integument has unusual biochemical characteristics. The increased staining of inner integumentary cells at fertilization in *E. ibaguense* (see Fig. 1c in Yeung 2022) draws attention to the special cytological features. When re-examining reports on orchid ovule development, increased staining intensity in the inner integument is often noted, e.g., *Oncidium flexuosum* (see Figs. 24–29 in Mayer et al. 2011), *Acianthera johannensis* (see Fig. 5 in Duarte et al. 2019) and *Dendrobium nobile* (see Figs. 4 e and f in Kolomeitseva et al. 2021). The inner integument of *Calypso bulbosa* shows a higher staining intensity until the suspensor begins to extend beyond it (see Figures in Yeung and Law 1992). In *Liparis parviflora* (see Fig. 1 in Kolomeitseva et al. 2019), the inner integument gives a strong autofluorescence at fertilization. Although the exact function is unknown, the biochemical and cytological features indicate that the integument can play an important role during fertilization and proembryo development. As discussed by Yeung (2022), since an endosperm fails to form, could the inner integument function as an "endosperm substitute" in orchid seeds during early embryo development?

It is well established that auxin is a crucial player in embryogenesis. Recently, Robert et al. (2018) demonstrated that the integuments are the source of auxin, regulating embryo morphogenesis in *Arabidopsis*. In the asexual race of *Spiranthes cernua*, cells of the inner integument, especially those at the tip of the micropyle, become highly cytoplasmic and develop into adventive embryos (Swamy 1948). In the *Zeuxine strateumatica* complex, adventive embryos can arise from the nucellar epidermis or inner integument (Vij et al. 1982). Judging from increased staining intensity and metabolic activities of the inner integument, plant growth substances could be one type of product produced, generating added morphogenetic potential.

The funiculus is thin, with no vascular elements connecting the developing ovules and seeds to the placenta (Figs. 1F, 2D, and 3F). In an ovule, nutrients are transported in a symplastic manner through plasmodesmata from the chalaza to the embryo sac. Although the translocation path is shorter from the hypostase/postament to the embryo sac, a longer route is preferred. In *Vanilla*, the fluorescent marker uranin is transported to the micropylar end along the inner integument before the appearance of fluorescence in the egg apparatus (Zhang and Zheng 1988). Together with the cytological features of the cells, the inner integument could have enhanced nutrient transfer ability, especially at the micropylar end, where the proembryo develops after fertilization.

The micropyle is a unique and common feature of an ovule; it serves as the entry point for the pollen tube during fertilization. In flowering plants, the micropyle is organized by the contribution of both integuments. Even though the orchid ovules are usually bitegmic, the micropyle is often organized by the inner integument alone. This feature is noted in a majority of orchids, as reported in *Amitostigma kinishitae* (Abe 1977), *Herminium monorchis* (Fredrikson 1990), *Microstylis wallichii* (Sood and Rao 1986), and *Neuwiedia veratrifolia* (Gurudeva 2019). Histologically, the inner integument becomes multilayered, forming a prominent extension at the micropyle. The cells have a dense cytoplasm. With the numerous ovules present, can the micropylar integumentary cells play a role in attracting the pollen tubes to the ovules and aid in the fertilization process?

In the study of orchid ovule development, although descriptive accounts of integument formation are available, the potential functions of the integuments are seldom discussed. We hope to draw attention to the importance of the integuments in ovule and proembryo development and encourage more focused studies of this tissue in the future.

## Seed coat development and structural features

After fertilization, the integuments develop into the seed coat. The nucellus disintegrates at the time of embryo sac maturation or soon after. The inner integument usually fails to develop further, becomes compressed, and collapses over the expanding embryo. Hence, the seed coat is derived mainly from the funiculus, chalaza, and outer integumentary cells in a mature orchid seed.

Cells of the outermost layer of the seed coat are lignified, usually at the radial walls and the inner tangential walls. The subepidermal thin-walled layer(s) subsequently collapsed, resulting in seeds having a single layer of seed coat cells. Moreover, in some terrestrial species such as *Dactylorhiza majalis* (Rasmussen 1995), *Epipactis* (Additional file 2), *Cypripedium formosanum* (Figs. 5 and 6) (Lee et al. 2005), and *Cypripedium plectrochilum* (Fig. 7), the inner integument remains alive with the ability to synthesize and accumulate lipidic and phenolic compounds before the cells collapse over the embryo. This additional covering is termed the 'carapace,' a protective shield (Veyret 1969; Rasmussen 1995), contributing to the added embryo protection. The following examples document the development of seed coats with different structural organizations.

### Orchid seeds with a single layer of seed coat cells at maturity and without a carapace

In *E. ibaguense*, the fertilized ovules undergo rapid enlargement and elongation along the length of the funiculus-chalaza. The inner integumentary tissue is ruptured and destroyed with the rapid growth of the embryo proper (Fig. 1F, G). As a result, the cells of the inner integument appear as remnants adhering to the embryo proper, within the seed cavity. Hence, the mature seed coat forms from the outer integumentary tissue only.

As the embryo develops, the suspensor elongates towards the tip of the micropyle formed by the outer integument. The suspensor is in close contact with the inner cells of the seed coat, especially on the funiculus side (Fig. 1H). These seed coat cells remain thin-walled and not lignified, as judged by the purple color of the TBO stain. During the early stages of seed development, these thin-walled cells remain alive, as indicated by a nucleus within cells (Fig. 1H). As the embryo matures, the suspensor and the thin seed coats become dried and difficult to be discerned.

A cavity is often noted in orchid seeds, especially at the chalazal end of the seed. The air inside the seed coat makes the seeds buoyant and readily dispersed. In *E. ibaguense*, the inner seed coat cells in the chalaza region fail to divide further after fertilization. With fewer cells and continual elongation of the outer layers, the inner cells separate and disintegrate, forming a chalazal cavity (Fig. 1I). In a mature seed, cell remnants suspend the embryo in this air-filled cavity. Lignification of the outermost layer of the seed coat cells begins early, with lignin deposition occurring in the radial walls and inner tangential wall while the outer walls remain thin (Fig. 1J). Moreover, lignified outer tangential walls can be seen in some mature seed coat cells near the embryo proper. Only a single lignified seed coat encloses the embryo at the time of seed maturation.

A similar pattern can be found in *P. tankervilliae* (Fig. 2). The expanding embryo cavity results in the compression and collapse of the inner integument (Fig. 2E). The expanding suspensor protrudes beyond the micropyle. It grows towards the outer opening delimited by the outer integument (Fig. 2F, Ye et al. 1997). Like *E. ibaguense*, the suspensor is in close contact with the seed coat cells, which are not lignified as judged by the staining reaction towards the TBO. These inner seed coat cells remain thin-walled and alive (Fig. 2G) before embryo maturation. In *P. tankervilliae*, lignification of the seed coat's outermost layer begins before embryo maturation. The radial and inner tangential walls show secondary thickenings (Fig. 2H). At maturity, all inner thin-walled seed coat cells have collapsed, partially covering the embryo (Fig. 2I). Thus, a mature seed coat is comprised of only a single layer of cells (Fig. 2I).

In the above examples, the behavior of the suspensor influences the final seed coat structure. The rapid growth of the suspensor and the increased size of the embryo prevent further development of the inner integument into an integral structural component of the mature seed coat.

### Orchid seed coat with a carapace

In several *Cypripedium* species, besides having an outer seed coat, the mature embryo is covered by a tight thin layer which has been called "carapace" (Figs. 5H, I) (Lee et al. 2005, 2015). In *C. formosanum*, the ovule's inner integument forms the carapace. The inner integument appears as a small projection at the base of the nucellar filament during archesporial cell formation (Fig. 5A). As the megaspore undergoes meiosis, the inner integument continues to extend toward the tip of the nucellar filament (Fig. 5B). It eventually encloses the developing embryo sac (Fig. 5C). After fertilization, the embryo cavity enlarges slightly after fertilization and remains the same till seed maturation. Mitotic activity is not detected within the inner integument (Fig. 5D). As the seed approaches maturity, the cells of the inner integument begin to dehydrate and compress into a tight thin layer (Fig. 5E–G), wrapping around the embryo. It stains blue with the TBO stain and reacts positively to Nile red stain, indicating the presence of lignin and cuticular substances, respectively (Fig. 6A, B). It is important to note that the embryo of *C*. *formosanum* has a short, single-celled suspensor (Fig. 5F). It is not a haustoria-like suspensor similar to *E. ibaguense* and *P. tankervilliae*.

The structural features of carapace vary among species. In *C. plectrochilum*, a distinct carapace is formed during seed development. A thin transparent seed coat houses the color carapace derived from the inner integument, which covers the mature embryo (Fig. 7). Similar to *C. formosanum*, cuticular substance, and lignin is present in the carapace cell walls. In addition, phenolic substances are synthesized and fill the vacuole of the cells (Fig. 7A). This gives the seeds an orange-black color. At maturity, the carapace shrinks, forming a distinct and thick layer wrapping around the embryo (Fig. 7B).

### Orchid seed with a multilayered seed coat and the presence of a carapace

The *Vanilla* seeds differ morphologically and structurally from other orchid seeds. The seed coat is sclerotic, a feature seldom found in orchid seeds (Fig. 3I). In *V. planifolia*, two distinct seed coat layers surround the embryo during seed development (Figs. 3E and 8A).

The inner seed coat, derived from the inner integument, is two cells thick, and the walls remain primary during the early stages of seed development (Fig. 8A, B). As the seeds approach maturity, the inner seed coat becomes gradually compressed and eventually forms a thin layer at maturity covering the embryo, creating a carapace (Fig. 8C, D). Using the Nile red staining, the inner seed coat's innermost and outermost surface walls react positively, indicating the possible accumulation of a cuticular substance in the wall of these cell layers (see micrographs in Yeh et al. 2021).

The outer integument is responsible for forming the seed coat. Before fertilization, the outer integument is still growing (Fig. 3B, C). It has not enclosed the embryo sac completely. At this stage, the walls of the outer seed coat cell are relatively thin (Fig. 3C). After fertilization, the walls of the outermost layer of the seed coat become thickened (Fig. 3D–F). As the embryo becomes matured, the thickened walls of the outer seed coat become sclerified (Fig. 3G, H). At the same time, the dark material accumulates further in the outer and lateral walls of the outermost cell layer (Fig. 8B, C). The thickened cell walls with dark material occupy the entire cell cavity, and the cells become sclerotic as the embryo matures (Figs. 3I and Fig. 8D). Near maturity, the inner layers of the outer seed coat gradually compress and attach to the sclerified outermost layer of the seed coat (Fig. 8C, D). Using the TBO stain, the cell wall of the outermost layer of the seed coat stained greenish-blue, indicating the presence of lignin in the wall.

The fruits and seeds in *Vanilla* species are designed for zoochory (Nishimura and Yukawa 2010; Pansarin and Ferreira 2021). The fruits and the sclerotic seeds in *Vanilla* are intended to be eaten by birds or other animals as the fresh fruits turn red as they mature, and birds are confirmed to be the primary seed dispersal agent. The digestive enzyme of birds sclerifies the hard seed coats, breaking dormancy and promoting germination (Nishimura and Yukawa 2010; Pansarin and Ferreira 2021; Zhang et al. 2021).

### Seed coat from unitegmic ovules

The unitegmic ovule is found in some mycoheterotrophic orchids, e.g., *Gastrodia* and *Epipogium* (Tohda 1967; Abe 1976; Arekal and Karanth 1981; Li et al. 2016). In these species, the seed coat comprises a single integument with only two cells thick. During the seed development of *G. nantoensis*, the seed coat cells become more vacuolated and enlarge further, and the starch grains are metabolized as the seed matures (Fig. 4E–G). At maturity, seed coat cells eventually compress into a thin layer and envelop the embryo (Fig. 4H). In *G. nantoensis*, the compressed thin seed coat stains greenish blue with the TBO stain, indicating the lignified cell wall (Fig. 4H). The seed coat also reacts weakly to the Nile red staining (Fig. 4I). Still, the signals could be easily quenched by pre-staining of TBO, indicating the absence of distinct cuticular materials. The fruiting period of *Gastrodia* and *Epipogium* is relatively short compared to most orchids; their above-ground parts last only 3–4 weeks, then vanish (Arekal and Karanth 1981). Since *Gastrodia* and *Epipogium* are fully mycoheterotrophic species that rely entirely on the nutrient supply from mycorrhizal fungi (Yagame et al. 2007; Li et al. 2016), the seed coat's simple structure may help reduce nutrient investment during reproduction.

### The characteristics of lignin and cutin deposits in the orchid seed coat

The seed coat is the first protective barrier against environmental stresses such as moisture and pathogens (Mohamed-Yasseen et al. 1994; Rajjou and Debeaujon 2008). In addition to the cellulosic walls, different polymers can be found embedded or encrusted in the seed coat cell walls, i.e., lignin, suberin, and cutin (Sano et al. 2016). These compounds can offer additional protection and reinforce the walls. In the orchid seed coat, lignification of seed coat cells appears universal, and its presence is deemed essential in its ability to protect the embryo within.

Lignin is readily identified using histochemical tests, i.e., phloroglucinol-HCl and TBO, and autofluorescence characteristics when viewed with a fluorescence microscope. Modern techniques such as vibrational spectroscopy and nuclear magnetic resonance provide vigorous methods for identifying lignin and studying its chemistry (Lupoi et al. 2015). Barsberg et al. (2013) confirm the presence of lignin in *Cypripedium calceolus* using FT-IR spectroscopy. In recent years, a new form of lignin, the C-lignin, was discovered by nuclear magnetic resonance (NMR) spectroscopy in seed coats of certain species belonging to Orchidaceae and Cactaceae (Chen et al. 2012, 2013; see Barsberg et al. 2018). The C-lignin differs from the commonly known G/S lignin because it is synthesized from caffeyl alcohol. Using the ATR-FT-IR spectroscopy, Barsberg et al. (2018) characterized seed coat ontogenesis and chemistry in three orchid species, i.e., *Neuwiedia veratrifolia, C. formosanum*, and *Phalaenopsis aphrodite* and discuss C-lignin properties and possible function to seed coat properties. They revealed and noted the marked diversity with respect to the seed surface chemistry of the orchids studied. Future investigations will provide further insight and possible implications for seed ecology and germination (Barsberg et al. 2018).

The presence of a cuticle is a common feature in many seed coats, e.g., cotton (Yan et al. 2009) and soybean (Ranathunge et al. 2010). The accumulation of cuticular material is commonly observed in the epidermal tissue, forming a vital hydrophobic barrier over the aerial surfaces, preventing water loss and gaseous exchanges (Esau 1977). In recent years, Nile red, a sensitive lipid stain (Greenspan et al. 1985), is often used to detect lipidic substances on the surface of epidermal cells and the embryo and has contributed to the characterization of cuticular substances in plant cell walls.

In orchids, the deposition of cuticular substances in orchid seed coats varies among species, and a distinct cuticle is absent in the seed coat walls. A lipid component is not detected using the lipid stain, Sudan III (Carlson 1940), and the IR spectroscopic method (Barsberg et al. 2013) in the *C. parviflorum* and *C. calceolus* seed coat*,* respectively. *Cyrtosia javanica,* a mycoheterotrophic orchid species, has a thick seed coat from the outer integument (see micrographs in Yang and Lee 2014). The outermost layer is sclerified with thick lignified walls. However, Nile red staining fails to detect the presence of a lipidic substance in the outer seed coat layers. Moreover, weak positive staining is found in the walls of the inner seed coat cells derived from the inner integument (Yang and Lee 2014). A positive Nile red staining is noted in *Cymbidium sinense* (Yeung et al. 1996) and *C. formosanum* (Lee et al. 2005). However, the stain is quenched by prestaining with TBO, indicating that the cuticular substance is adcrusted in the wall and not as a distinct cuticle similar to that commonly seen in leaf epidermal cells.

Cutin deposits are more consistently found when a carapace is present and at the embryo's surface. In *Cephalanthera falcata*, lignin and cuticular material accumulation have been reported in the inner seed coat (Yamazaki and Miyoshi 2006). Similar intense staining of Nile red can be seen in the inner walls (carapace) derived from the inner integument in *C. formosanum* (Lee et al. 2005). Positive Nile red staining is often noted in the outer walls of the orchid embryos, such as *C. sinense* (Yeung et al. 1996) and *Paphiopedilum delenatii* (Lee et al. 2006). The presence of cuticular material offers additional protection to the embryo.

## Seed coat functions during seed development and germination in orchids

Due to the simplicity of the seed coat structures in orchids, besides aiding in seed dispersal and serving a protective function during seed germination, a discussion on its functions during development is absent from the literature. Here, we summarize current observations and draw attention to the seed coat's additional functions during development and germination.

### Nutrient supply during seed development

The inner layer of the seed coat derived from the outer integument is destined to aid in nutrient transfer to the developing embryo, especially when a haustoria-like suspensor is present. As shown in *E. ibaguense* and *P. tankervilliae*, the inner layers of the seed coat derived from the outer integument remain alive with thin walls during the early stages of embryo development. The walls stain purple with the TBO stain. This polychromatic stain can distinguish lignin, cellulose, and pectic substances based on color differences (O'Brien et al. 1964; O'Brien and McCully 1981). The purple-color reaction towards the TBO stain indicates the absence of phenolic compounds in the wall, which can impede the apoplastic transport process. Furthermore, the absence of autofluorescence and Nile red stain in these cell layers indicates the lack of lipidic and phenolic compounds in the walls (Yeung et al. 1996). These features enable the suspensor to obtain nutrients apoplastically through the walls of the seed coat and translocate them to the embryo proper. Our earlier study demonstrates that the suspensor cell of *P. tankervilliae* has a more negative osmotic potential than neighboring cells, providing a driving force for the uptake of water and nutrients from adjoining seed coat cells (Lee and Yeung 2010). A recent comparative study using suspensors of *Arabidopsis* and beans indicates that genes involved in transport and Golgi body organization are upregulated in the suspensor (Chen et al. 2021), indicating that the suspensor has unique physiological properties. By positioning itself next to the source of nutrients, i.e., the thin seed coat cells, nutrient acquisition for the embryo can be achieved.

### Carapace formation for added protection in seed dispersal and germination

The term carapace is defined as a protective shell. It originates from the inner integument and wraps around the embryo (Veyret 1969; Rasmussen 1995; Lee et al. 2005; Yamazaki and Miyoshi 2006). This structure is common in temperate, terrestrial orchids such as *Dactylorhiza* species (Custódio et al. 2016) and *Paphiopedilum* species (Lee et al. 2006). The thickness of the carapace varies. Synthesis and deposition of phenolic compounds occur before the inner integumentary cells collapse, offering further protection to the embryo. In *Cephalanthera falcata* (Yamazaki and Miyoshi 2006), a carapace is readily detected and wrapped tightly around the embryo. A thin carapace is seen in *C. formosanum* (Lee et al. 2005), while *Limodorum* (Veyret 1969) and *C. plectrochilum* (Fig. 7) have a relatively thick carapace.

From the case histories shown earlier, it is clear that a carapace cannot be formed in embryos with a haustoria-like suspensor. As seen in *E. ibaguense*, *P. tankervilliae* as well as *Phalaenopsis* (Additional file 3; Lee et al. 2008), the rapid elongation of the suspensor and the growth of the embryo tend to rupture the inner integument preventing carapace formation. Moreover, to fulfill a protective function, we propose that the term 'carapace' should be applied to those seeds with a distinct inner layer derived from the inner integument, having lipidic and or phenolic deposits incorporated in the cellulosic walls. The added compounds serve to provide added protection to the embryo in addition to the seed coat.

It is well established for in vitro seed germination that carapace is one of the major causes inhibiting mature seed germination. Veyret (1969) noted that seeds with a particularly well-developed carapace, such as *Cephalanthera* and *Epipactis* species (Additional file 2), germinated with difficulty. The carapace acts as a barrier to water and nutrient absorption. Sonification modifies the carapace through physical scarification and improves germination (Miyoshi and Mii 1988). Stratification of the seed coat using NaOCl improves tetrazolium staining in seeds with a thick carapace (Custodio et al. 2016). Seed pretreatment could improve seed coat hydrophilicity and permeability, allowing germination (Miyoshi and Mii 1998; Lee et al. 2007; Lee 2011; Šoch et al. 2023).

The presence of a carapace is important to the survival of orchid seeds in their natural environment. The carapace is more often found in seeds of temperate terrestrial orchids (Rasmussen 1995; Lee et al. 2005; Yamazaki and Miyoshi 2006)**.** Besides, functions as an additional protective layer, can the presence of a carapace result in seed coat-imposed dormancy, regulating seed germination in its natural habitat? In the temperate region, seeds are shed in the autumn. A carapace may protect the embryo, allowing the seeds to survive the winter months and delaying germination until spring.

### Seed coat features allow water uptake during germination

As indicated above, the inner seed coat layer cells have no phenolic deposits and will not pose as an apoplastic barrier for water movement during seed imbibition. Even though the cells have collapsed as the seeds dry, the walls can still serve as channels for the apoplastic movement of water and water-soluble materials during germination.

Particular structural adaptations for water uptake and storage have been noted in the seed coat of *Sobralia dichotoma* (Prutsch et al. 2000). The seed coat in *S. dichotoma* consists of different cell types, i.e., helical tracheoidal cells and collapsed cells with walls rich in pectin. Imbibition leads to uncoiling, stretching the helical tracheoidal cells forming a pipe, and shaping a central capillary. The reversible movement of the helical tracheoidal cells is interpreted as a mechanism of water uptake (uncoiling) and -storage (coiling). The pectin-rich cells may function in water storage, thereby protecting the mature embryo against desiccation. This intricate design demonstrates that orchid seed coat can have specialized functions even though cells are no longer alive.

### The varied thickened seed coat adapts to the seed dispersal mechanism

Although most orchids have a thin seed coat at maturity, orchids with fresh, colorful fruits and thick-walled seeds are designed for zoochory. As shown in *Apostasia nipponica* (Suetsugu 2020),* C. javanica* (Yang and Lee 2014), *Cyrtosia septentrionalis* (Suetsugu et al. 2015), *Neuwiedia singapureana* (Zhang et al. 2021) and *Yoania japonica* (Suetsugu 2018a, b), these species have fleshy fruits containing seeds with a thick seed coat. The thickened and lignified seed coat protects seeds from the digestive enzyme as they pass through the digestive tracts of birds. Moreover, the digestive process modifies the seed coat, enhancing germination (Zhang et al. 2021). A similar observation is well documented in *Vanilla* species (Pansarin and Ferreira 2021; Yeh et al. 2021). It is likely for those orchid species with fresh fruit and seeds, having a thick seed coat is an adaptation to their elected reproductive strategies. In their review, Coen and Magnani (2018) recently indicated, "the seed coat architecture evolved to adapt to different environment and reproductive strategies in part by modifying its thickness." The varied number of integuments and thickness of the seed coat found in orchid species are likely to be adaptive features for seed dispersal and germination.

### The seed coat directs the entry of fungal hyphae through the micropyle during symbiotic seed germination

For symbiotic seed germination, the successful penetration and establishment of a compatible mycorrhizal fungus into the embryo ensures protocorm formation. Although the seed coat structure is simple, its design is part of the strategy ensuring success. The prominent micropylar opening is a clever design providing an initial site of entry for the mycorrhizal fungi for most orchid species. In *Bletilla striata*, embryos with the seed coat removed result in a lower germination rate than intact seeds infected with appropriate symbiotic fungi (Miura et al. 2019). This finding indicates that restricting the invasion of fungal hyphae at the initial stage of fungal colonization allows proper symbiotic establishment. The entry of mycelium through the micropyle into the degenerated suspensor of the embryo is one of the preferred pathways (Yeung et al. 2019), ensures the 'planned' sequence of events, such as peloton formation, can occur, resulting in protocorm growth and development. In *Caladenia tentaculate*, the embryo produces a UV autofluorescing substance which gradually recedes towards the suspensor region near the micropyle (Wright et al. 2005). Although the nature and function are unknown, this substance may interact with compatible mycorrhizal fungi, establishing symbiotic interactions. It is important to note that even though the suspensor has degenerated, the absence of cuticular materials in its wall enables the ready penetration of mycelium into the embryo properly. The seed coat at the chalazal end can also accommodate the expansion of the embryo, forming a tight fit over the embryo at the chalazal end during the early stages of germination. This safeguards the entry of fungal hyphae into the embryo's future shoot apical zone, allowing proper shoot development. Moreover, the compatibility between the fungi and the orchids is a critical factor in determining the ultimate success of symbiotic seed germination (Chen et al. 2022). Although the function of the seed coat is 'passive', the structural design enables it to play a role in the early stages of symbiotic seed germination.

## Perspective

The orchid seed coat has a simple structure. The minute size of the seeds makes this a difficult experimental material to study. Moreover, their simple organization is likely an adaptation to reproductive strategies. This review draws attention to aspects of seed coat structures and their potential functions during seed development and germination. Moreover, many important questions remain. For example, does the seed coat have a morphogenetic role in embryo development without an endosperm besides nutrient supplies? In flowering plants, there is a close interplay between the endosperm and seed coat formation (Ingouff et al. 2006; Wang et al. 2021). Without endosperm, could a similar process occur between the orchid embryo and the seed coat?

With an improved appreciation of seed coat development and function, we can focus on studying key processes such as nutrient transfer between the seed coat and the embryo and the biosynthesis of secondary metabolites in carapace formation. Our understanding of the molecular control of seed coat development still has many gaps (Matilla 2019). Recently, a MADS-box gene, PeMADS28 has been identified in orchids in *Phalaenopsis equestris* and has been shown to play an essential role in ovule integument development (Shen et al. 2021). Is there a seed coat-specific promoter in orchids that regulates integument and seed coat development? More studies are needed on molecular genetics and gene functions during development. We also see the potential of the seed coat system in unraveling new regulatory mechanisms and providing new perspectives on plant biology. The recent successful use of the RNA-seq method with the laser microdissection technique described by Millar et al. (2015) and Balestrini et al. (2021) can provide precise answers to the question posted. With further refinement in cell isolation techniques, it would be possible to apply single-cell RNA sequencing technology (Xu and Jackson 2023) to study specific events in the integument and seed coat development.

### Supplementary Information

**Additional file 1. **The seed coat development of *Epipogium roseum*. **A** A longitudinal section through a mature embryo sac showing the egg apparatus. At this stage, the integument tissue (arrow) has not completely enclosed the nucellus (arrowhead). Scale bar = 50 μm**.** **B** At fertilization, the integument does not envelop the embryo sac (arrow). An arrowhead indicates the nucellus, and the double arrowhead indicates the degenerated synergid. Scale bar = 50 μm. **C** At the stage of a two-celled embryo, the seed coat (arrow) has enclosed the embryo sac completely. Starch grains appear within the cytoplasm of the embryo. The nucellus (arrowhead) is still distinct at this stage of development. Scale bar = 50 μm. **D** The two-celled embryo divides once, resulting in a four-celled embryo. More starch grains accumulate within the cytoplasm of the embryo. The cells of the seed coat (arrow) enlarge further, and the nucellus (arrowhead) begins to degenerate. Scale bar = 50 μm. **E** At maturity, the embryo is enveloped by a shriveled seed coat (arrow). Scale bar = 50 μm. **F** A mature seed of *E. roseum* takes on a pear-like form. Scale bar = 50 μm.

**Additional file 2. **The formation of carapace in *Epipactis mairei* seeds. **A** Light micrograph showing a Spurr's resin section through a maturing globular embryo after the TBO staining. Protein and lipid bodies are the main storage products within the embryo cells. The cells of the inner seed coat (arrowhead) begin to shrivel. Scale bar = 20 μm. **B** Light micrograph showing a Spurr's resin section through a mature seed after the Sudan Black B staining. The embryo proper is enveloped by the shriveled inner seed coat, i.e., the carapace (arrowhead), which is protected by a thin outer seed coat (arrow). Scale bar = 20 μm.

**Additional file 3. **The seed coat development of *Phalaenopsis aphrodite.* **A** After fertilization, the zygote (*) has a dense cytoplasm, and the inner seed coat (arrowhead) encloses the embryo sac completely. The outer seed coat (arrow) cells have expanded and elongated by vacuolation. Scale bar = 20 μm. **B** At the four-celled embryo stage, the two cells toward the chalazal end are small with dense cytoplasm, while the other two cells toward the micropylar end continue to enlarge. The cells of the inner seed coat (arrowheads) begin to condense and then degenerate. The cells of the outer seed coat (arrow) also begin to condense, but they still stay turgid. Scale bar = 20 μm. **C** At the early globular stage, the suspensor cells (S) have elongated and surround the developing embryo proper. The cells of the inner seed coat (arrow) have degenerated completely, and the radial walls of the outer seed coat (arrowhead) have become thickened. Scale bar = 20 μm. **D** At the globular stage, a cell size gradient is noted in the embryo proper, with smaller cells in the chalazal region and larger cells toward the micropylar end. The suspensor cells (S) and the cells of the outer seed coat (arrow) are undergoing dehydration and becoming shriveled. Scale bar = 20 μm. **E** As development progresses, the embryo cells become cytoplasmic with dense cytoplasm. The seed coat has shriveled (arrow). Scale bar = 20 μm. **F** At maturity, the embryo cells have an abundant reserves deposit. The embryo is enveloped by the shriveled seed coat (arrow). Scale bar = 20 μm

## Data Availability

Not applicable.

## References

[CR1] Abe K (1968). Contributions to the embryology of the Orchidaceae. III. Development of the embryo sac in *Cremastra appendiculata*. Sci Rep Tohoku Univ Ser IV (biol).

[CR2] Abe K (1971). Contributions to the embryology of the family Orchidaceae. V. Development of the embryo sac in *Oreorchis patens*. Sci Rep Tohoku Univ Ser IV (Biol).

[CR3] Abe K (1972). Contributions to the embryology of the family Orchidaceae. VII. A comparative study of the orchid embryo sac. Sci Rep Tohoku Univ Ser IV (Biol).

[CR4] Abe K (1976). A reinvestigation of the development of the embryo sac in *Gastrodia elata* Blume (Orchidaceae). Ann Bot.

[CR5] Abe K (1977). Development of the embryo sac in *Amitostigma kinoshitae* (Makino) Schltr. (Orchidaceae). Ann Bot.

[CR6] Afzelius K (1954). Embryo-sac development in *Epipogiwn aphyllum*. Svensk Bot Tidskr.

[CR7] Alvarez MR (1968). Temporal and spatial changes in peroxidase activity during fruit development in *Encyclia tampensis* (Orchidaceae). Am J Bot.

[CR8] Alves MF, Pinheiro F, Niedzwiedzki MP, Mayer JLS (2019). First record of ategmic ovules in Orchidaceae offers new insights into mycoheterotrophic plants. Front Plant Sci.

[CR9] Aprilianti P, Handini E, Puspitaningtyas DM (2021). A seed morphometry study of selected species of *Bulbophyllum* and *Dendrobium* (Orchidaceae) in relation to their dispersals. Biodiversitas.

[CR10] Arditti J, Ghani AKA (2000). Numerical and physical properties of orchid seeds and their biological implications. New Phytol.

[CR11] Arekal GD, Karanth KA (1981). The embryology of *Epipogium roseum* (Orchidaceae). Plant Syst Evol.

[CR12] Balestrini R, Perotto S, Fiorilli V (2021). Laser microdissection as a tool to study fungal gene expression in mycorrhizal endosymbiosis. Italian J Mycology.

[CR13] Barsberg S, Rasmussen HN, Kodahl N (2013). Composition of *Cypripedium calceolus* (Orchidaceae) seeds analyzed by attenuated total reflectance IR spectroscopy: in search of understanding longevity in the ground. Amer J Bot.

[CR14] Barsberg ST, Lee YI, Rasmussen HN (2018). Development of C-lignin with G/S-lignin and lipids in orchid seed coats–an unexpected diversity exposed by ATR-FT-IR spectroscopy. Seed Sci Res.

[CR15] Barthlott W, Große-Veldmann B, Korotkova N (2014). Orchid seed diversity: a scanning electron microscopy survey.

[CR16] Boesewinkel FD, Bouman F, Kigel J, Galili G (1995). The seed: structure and function. Seed Development and Germination.

[CR17] Carlson MC (1940). Formation of the seed of *Cypriedium parviflorum*. Bot Gaz.

[CR18] Chaban IA, Gulevich AA, Kononenko NV, Khaliluev MR, Baranova EN (2022). Morphological and structural details of tomato seed coat formation: a different functional role of the inner and outer epidermises in unitegmic ovule. Plants.

[CR19] Chen F, Tobimatsu Y, Havkin-Frenkel D, Dixon RA, Ralph J (2012). A polymer of caffeyl alcohol in plant seeds. Proc Natl Acad Sci USA.

[CR20] Chen F, Tobimatsu Y, Jackson L, Nakashima J, Ralph J, Dixon RA (2013). Novel seed coat lignins in the Cactaceae: structure, distribution and implications for the evolution of lignin diversity. Plant J.

[CR21] Chen M, Lin JY, Wu X, Apuya NR, Henry KF, Le BH (2021). Comparative analysis of embryo proper and suspensor transcriptomes in plant embryos with different morphologies. Proc Natl Acad Sci USA.

[CR22] Chen X-G, Wu Y-H, Li N-Q, Gao J-Y (2022). What role does the seed coat play during symbiotic seed germination in orchids: an experimental approach with Dendrobium officinale. BMC Plant Biol.

[CR23] Coen O, Magnani E (2018). Seed coat thickness in the evolution of angiosperms. Cell Molec Life Sci.

[CR24] Collier MH, Fisher JS, Gribbins KM, Yoder JA, Zettler LW (2023). Differences in seed morphometrics of representative orchids native to North America and Hawaii using scanning electron microscopy. S Afri J Bot.

[CR25] Custódio CC, Marks TR, Pritchard HW, Hosomi ST, Machado-Neto NB (2016). Improved tetrazolium viability testing in orchid seeds with a thick carapace (*Dactylorhiza fuchsii*) or dark seed coat (*Vanda curvifolia*). Seed Sci and Technol.

[CR26] Dressler RL (1993). Phylogeny and classification of the orchid family.

[CR27] Duarte MO, Oliveira DMT, Borba EL (2019). Ontogenesis of ovary and fruit of *Acianthera johannensis* (Pleurothallidinae, Orchidaceae) reveals a particular female embryology. Flora.

[CR28] Esau K (1977). Anatomy of seed plants.

[CR29] Fredrikson M (1990). Embryological study of *Herminium monorchis* (Orchidaceae) using confocal scanning laser microscopy. Am J Bot.

[CR30] Gamarra R, Dorda E, Scrugli A, Galán P, Ortúñez E (2007). Seed micromorphology in the genus *Neotinea* Rchb. f. (Orchidaceae, Orchidinae). Bot J Linnean Soc.

[CR31] Greenspan P, Mayer EP, Fowler SD (1985). Nile red: a selective fluorescent stain for intracellular lipid droplets. J Cell Biol.

[CR32] Gurudeva MR (2019). Ontogeny and organization of female gametophyte in triandrous orchid, *Neuwiedia veratrifolia* Blume (Orchidaceae) - A re-investigation. J Orchid Soc India.

[CR33] Hariyanto S, Pratiwi IA, Utami ESW (2020). Seed morphometry of native Indonesian orchids in the genus *Dendrobium*. Scientifica.

[CR34] Ingouff M, Jullien PE, Berger F (2006). The female gametophyte and the endosperm control cell proliferation and differentiation of the seed coat in *Arabidopsis*. Plant Cell.

[CR35] Kodahl N, Johansen BB, Rasmussen FN (2015). The embryo sac of *Vanilla imperialis* (Orchidaceae) is six-nucleate, and double fertilization and formation of endosperm are not observed. Bot J Linnean Soc.

[CR36] Kolomeitseva GL, Ryabchenko AS, Babosha AV (2019). The first stages of *Liparis parviflora* (Orchidaceae) embryogenesis. Russian J Devel Biol.

[CR37] Kolomeitseva GL, Babosha AV, Ryabchenko AS, Tsavkelova EA (2021). Megasporogenesis, megagametogenesis, and embryogenesis in *Dendrobium nobile* (Orchidaceae). Protoplasma.

[CR38] Kusano S (1915). Experimental studies on the embryonal development in an angiosperm. J Coll Agric Imp Univ Tokyo.

[CR39] Law SK, Yeung EC (1989). Embryology of *Calypso bulbosa*. I Ovule Development Amer J Bot.

[CR40] Lee YI, Thorpe TA, Yeung EC (2011). *In vitro* culture and germination of terrestrial Asian orchid seeds. Plant embryo culture: methods and protocols.

[CR41] Lee YI, Yeung EC (2010). The osmotic property and fuorescent tracer movement of developing orchid embryos of *Phaius tankervilliae* (Aiton) BI. Sex Plant Reprod.

[CR42] Lee YI, Lee N, Yeung EC, Chung MC (2005). Embryo development of *Cypripedium formosanum* in relation to seed germination *in vitro*. J Amer Soc Hort Sci.

[CR43] Lee YI, Yeung EC, Lee N, Chung MC (2006). Embryo development in the lady's slipper orchid, *Paphiopedilum delenatii* with emphases on the ultrastructure of the suspensor. Ann Bot.

[CR44] Lee YI, Lu CF, Chung MC, Yeung EC, Lee N (2007). Developmental changes in endogenous abscisic acid concentrations and asymbiotic seed germination of a terrestrial orchid, *Calanthe tricarinata* Lindl. J Am Soc Hortic Sci.

[CR45] Lee YI, Yeung EC, Lee N, Chung MC (2008). Embryology of *Phalaenopsis amabilis* var. *formosa*: embryo development. Bot Stud.

[CR46] Lee YI, Chung MC, Yeung EC, Lee N (2015). Dynamic distribution and the role of abscisic acid during seed development of a lady's slipper orchid, *Cypripedium formosanum*. Ann Bot.

[CR47] Li Y, Chen X, Guo S, Lee YI (2016). Embryology of two mycoheterotrophic orchid species, *Gastrodia elata* and *Gastrodia nantoensis*: Ovule and embryo development. Bot Stud.

[CR48] Lupoi JS, Singh S, Parthasarathi R, Simmons BA, Henry RJ (2015). Recent innovations in analytical methods for the qualitative and quantitative assessment of lignin. Renew Sustain Energy Rev.

[CR49] Matilla AJ (2019). Seed coat formation: its evolution and regulation. Seed Sci Res.

[CR50] Mayer JLS, Carmello-Guerreiro SM, Appezzato-da-Glória B (2011). Anatomical development of the pericarp and seed of *Oncidium flexuosum* Sims (Orchidaceae). Flora.

[CR51] Millar JL, Becker MG, Belmonte MF, Yeung EC, Stasolla C, Sumner MJ, Huang BQ (2015). Laser microdissection of plant tissues. Plant microtechniques and protocols.

[CR52] Miura C, Saisho M, Yagame T, Yamato M, Kaminaka H (2019). *Bletilla striata* (Orchidaceae) seed coat restricts the invasion of fungal hyphae at the initial stage of fungal colonization. Plants.

[CR53] Miyoshi K, Mii M (1988). Ultrasonic treatment for enhancing seed germination of terrestrial orchid, *Calanthe discolor*, in asymbiotic culture. Sci Hortic.

[CR54] Miyoshi K, Mii M (1998). Stimulatory effects of sodium and calcium hypochlorite, pre-chilling and cytokinins on the germination of *Cypripedium macranthos*. Physiol Plant.

[CR55] Mohamed-Yasseen Y, Barringer SA, Splittstoesser WE, Costanza S (1994). The role of seed coats in seed viability. Bot Rev.

[CR56] Moise JA, Han S, Gudynaite-Savitch L, Johnson DA, Miki BLA (2005). Seed coats: Structure, development, composition, biotechnology. In Vitro Cell Dev Pl.

[CR57] Molvray M, Kores PJ (1995). Character analysis of the seed coat in Spiranthoideae and Orchidoideae, with special reference to the Diurideae (Orchidaceae). Amer J Bot.

[CR58] Molvray M, Chase MW, Pridgeon AM, Cribb PJ, Chase MW, Rasmussen FN (1999). Seed morphology. Genera Orchidacearum Volume 1 General introduction, Apostasioideae, Cypripedioideae.

[CR59] Nishimura G, Yukawa T (2010). Dark material accumulation and sclerotization during seed coat formation in *Vanilla planifolia* Jacks. Ex Andrews (Orchidaceae). Bull Natl Mus Nat Sci Ser B.

[CR60] O'Brien TP, McCully ME (1981). The study of plant structure: principles and selected methods.

[CR61] O'Brien TP, Feder N, McCully M (1964). Poly-chromatic staining of plant cell walls by toluidine blue O. Protoplasma.

[CR62] Pansarin ER, Ferreira AWC (2021). Unravelling the enigma of seed dispersal in *Vanilla* (Orchidaceae). Plant Biol.

[CR63] Prutsch J, Schardt A, Schill R (2000). Adaptations of an orchid seed to water uptake and -storage. Plant Syst Evol.

[CR64] Radchuk V, Borisjuk L (2014). Physical, metabolic and developmental functions of the seed coat. Front Plant Sci.

[CR65] Rajjou L, Debeaujon I (2008). Seed longevity: survival and maintenance of high germination ability of dry seeds. CR Biol.

[CR66] Ranathunge K, Shao S, Qutob D, Gijzen M, Peterson CA, Bernards MA (2010). Properties of the soybean seed coat cuticle change during development. Planta.

[CR67] Rasmussen FN (1995). Terrestrial orchids – from seed to mycotrophic plant.

[CR68] Raviv B, Aghajanyan L, Granot G, Makover V, Frenkel O, Gutterman Y, Grafi G (2017). The dead seed coat functions as a long-term storage for active hydrolytic enzymes. PLoS ONE.

[CR69] Ren L, Wang FX (1987). Embryological studies of *Paphiopedilium godefroy* AE Stein. Acta Bot Sin.

[CR70] Robert HS, Park C, Gutièrrez CL, Wojcikowska B, Pěnčík A, Novák O, Chen J, Grunewald W, Dresselhaus T, Friml J, Laux T (2018). Maternal auxin supply contributes to early embryo patterning in *Arabidopsis*. Nat Plants.

[CR71] Sano N, Rajjou L, North HM, Debeaujon I, Marion-Poll A, Seo M (2016). Staying alive: Molecular aspects of seed longevity. Plant Cell Physiol.

[CR72] Shen CY, Chen YY, Liu KW, Lu HC, Chang SB, Hsiao YY, Yang F, Zhu G, Zou SQ, Huang LQ, Liu ZJ, Tsai WC (2021). Orchid B_sister_ gene PeMADS28 displays conserved function in ovule integument development. Sci Rep.

[CR73] Šoch J, Šonka J, Ponert J (2023). Acid scarification as a potent treatment for an in vitro germination of mature endozoochorous *Vanilla planifolia* seeds. Bot Stud.

[CR74] Sood SK, Rao PRM (1986). Gametophytes, embryogeny and pericarp of *Microstylis wallichii* Lindl. (Orchidaceae). Bot Mag Tokyo.

[CR75] Suetsugu K (2018). Independent recruitment of a novel seed dispersal system by camel crickets in achlorophyllous plants. New Phytol.

[CR76] Suetsugu K (2018). Seed dispersal in the mycoheterotrophic orchid *Yoania japonica*: further evidence for endozoochory by camel crickets. Plant Biol.

[CR77] Suetsugu K (2020). A novel seed dispersal mode of *Apostasia nipponica* could provide some clues to the early evolution of the seed dispersal system in Orchidaceae. Evolution Letters.

[CR78] Suetsugu K, Kawakita A, Kato M (2015). Avian seed dispersal in a mycoheterotrophic orchid *Cyrtosia septentrionalis*. Nature Plants.

[CR79] Swamy BGL (1947). On the life history of *Vanilla planifolia*. Bot Gaz.

[CR80] Swamy BGL (1948). Agamospermy in Spiranthes Cemua Lloydia.

[CR81] Tohda H (1967). An embryological study of *Hetaeria shikokiana*, a saprophytic orchid in Japan. Sci Rep Tohoku Univ Ser IV (biol).

[CR82] Veyret Y (1969). La structure des semences des *Orchidaceae* et leur aptitude à la germination in vitro en cultures pures. Travaux du Laboratorie de la Jaysinia.

[CR83] Vij SP, Sharma M, Shekhar N (1982). Embryological studies in Orchidaceae. II: *Zeuxine strateumatica* complex. Phytomorphology.

[CR84] Wang W, Xiong H, Sun K, Zhang B, Sun MX (2021). New insights into cell-cell communications during seed development in flowering plants. J Integrative Biol.

[CR85] Wright M, Guest D, Cross R (2005). Development of mycorrhiza association in *Caladenia tentaculata*. Selbyana.

[CR86] Xu X, Jackson D (2023). Single-cell analysis opens a goldmine for plant functional studies. Curr Opin Biotech.

[CR87] Yagame T, Yamato M, Mii M, Suzuki A, Iwase K (2007). Developmental processes of achlorophyllous orchid, *Epipogium roseum*: from seed germination to flowering under symbiotic cultivation with mycorrhizal fungus. J Plant Res.

[CR88] Yamazaki J, Miyoshi K (2006). *In vitro* asymbiotic germination of immature seed and formation of protocorm by *Cephalanthera falcata* (Orchidaceae). Ann Bot.

[CR89] Yan H, Hua Z, Qian G, Wang M, Du G, Chen J (2009). Effect of cutinase on the degradation of cotton seed coat in bio-scouring. Biotechnol Bioprocess Eng.

[CR90] Yang CK, Lee YI (2014). The seed development of a mycoheterotrophic orchid. Cyrtosia Javanica Blume Bot Stud.

[CR91] Ye XL, Zee SY, Yeung EC (1997). Suspensor development in the nun orchid, *Phaius tankervilliae*. Int J Plant Sci.

[CR92] Yeh CH, Chen KY, Lee YI (2021). Asymbiotic germination of *Vanilla planifolia* in relation to the timing of seed collection and seed pretreatments. Bot Stud.

[CR93] Yeung EC (2022). The orchid embryo - 'an embryonic protocorm'. Botany.

[CR94] Yeung EC, Law SK (1989). Embryology of *Epidendrum ibaguense*. I Ovule Development Can J Bot.

[CR95] Yeung EC, Law SK (1992). Embryology of *Calypso bulbosa*. II Embryo Development Can J Bot.

[CR96] Yeung EC, Law SK, Arditti J, Pridgeon AM (1997). Ovule development. Orchid biology: reviews and perspectives VII.

[CR97] Yeung EC, Zee SY, Ye XL (1996). Embryology of *Cymbidium sinense*: embryo development. Ann Bot.

[CR98] Yeung EC, Li YY, Lee YI (2019). An overview of the life of an orchid protocorm—a developmental perspective. Acta Hortic.

[CR99] Zhang ZJ, Zheng GJ (1988). Translocation of uranin within the living ovules of *Vanilla*. Acta Bot Sinica.

[CR100] Zhang Y, Li YY, Wang M, Liu J, Luo F, Lee YI (2021). Seed dispersal in *Neuwiedia singapureana*: novel evidence for avian endozoochory in the earliest diverging clade in Orchidaceae. Bot Stud.

[CR101] Zinger NV, Poddubnaya-Arnoldi VA (1966). Application of histochemical techniques to the study of embryonic processes in certain orchids. Phytomorphology.

