# Supplementary figures and images for: The orchid seed coat: a developmental and functional perspective
Source: Bot Stud. 2023 Sep 27;64:27. doi: 10.1186/s40529-023-00400-0 (PMC10533777; doi:10.1186/s40529-023-00400-0)

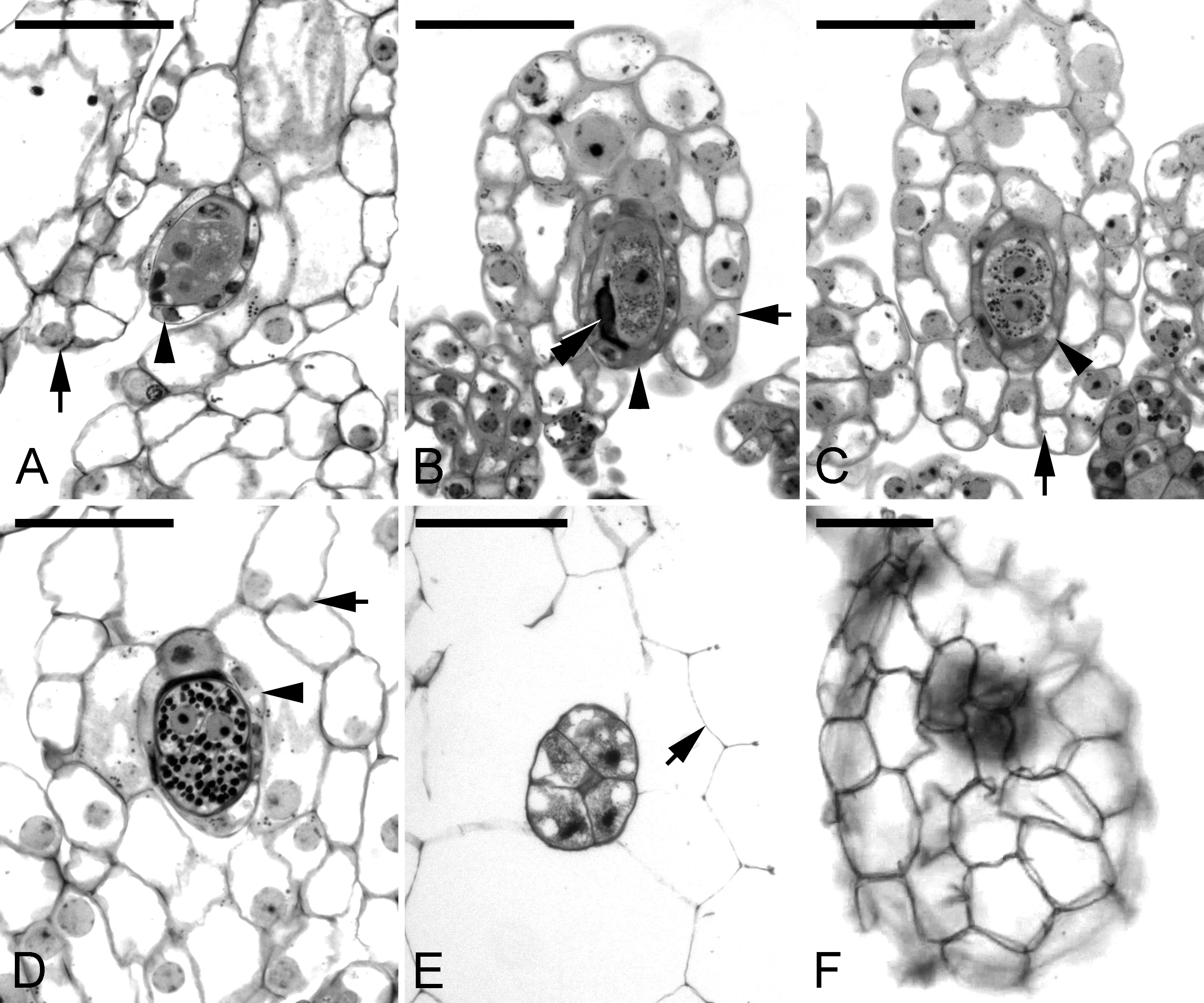

Supplement: Supplementary file 1 — Additional file 1. The seed coat development of Epipogium roseum. A A longitudinal section through a mature embryo sac showing the egg apparatus. At this stage, the integument tissue (arrow) has not completely enclosed the nucellus (arrowhead). Scale bar = 50 μm. B At fertilization, the integument does not envelop the embryo sac (arrow). An arrowhead indicates the nucellus, and the double arrowhead indicates the degenerated synergid. Scale bar = 50 μm. C At the stage of a two-celled embryo, the seed coat (arrow) has enclosed the embryo sac completely. Starch grains appear within the cytoplasm of the embryo. The nucellus (arrowhead) is still distinct at this stage of development. Scale bar = 50 μm. D The two-celled embryo divides once, resulting in a four-celled embryo. More starch grains accumulate within the cytoplasm of the embryo. The cells of the seed coat (arrow) enlarge further, and the nucellus (arrowhead) begins to degenerate. Scale bar = 50 μm. E At maturity, the embryo is enveloped by a shriveled seed coat (arrow). Scale bar = 50 μm. F A mature seed of E. roseum takes on a pear-like form. Scale bar = 50 μm. [file 40529_2023_400_MOESM1_ESM.jpg]

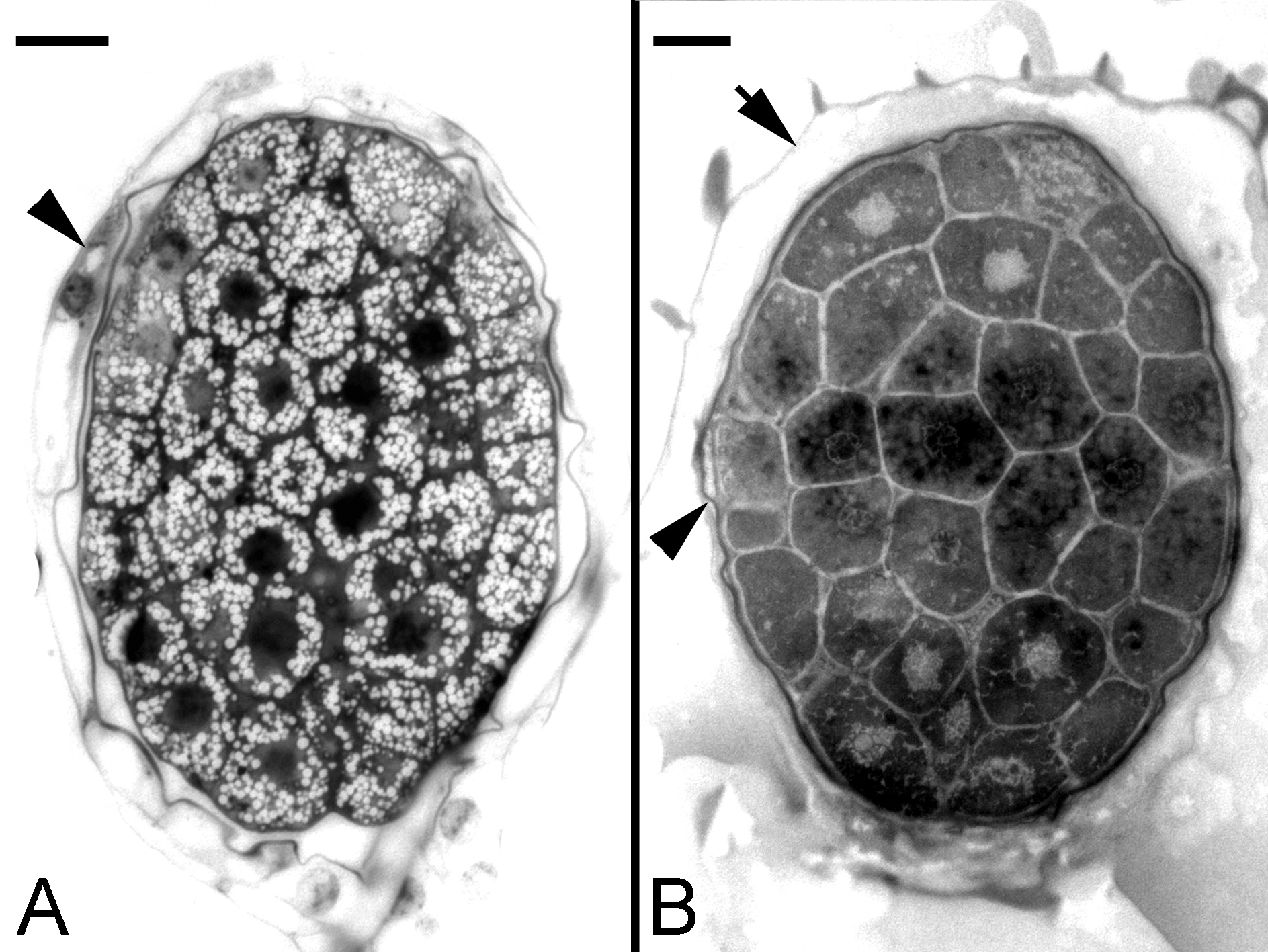

Supplement: Supplementary file 2 — Additional file 2. The formation of carapace in Epipactis mairei seeds. A Light micrograph showing a Spurr's resin section through a maturing globular embryo after the TBO staining. Protein and lipid bodies are the main storage products within the embryo cells. The cells of the inner seed coat (arrowhead) begin to shrivel. Scale bar = 20 μm. B Light micrograph showing a Spurr's resin section through a mature seed after the Sudan Black B staining. The embryo proper is enveloped by the shriveled inner seed coat, i.e., the carapace (arrowhead), which is protected by a thin outer seed coat (arrow). Scale bar = 20 μm. [file 40529_2023_400_MOESM2_ESM.jpg]

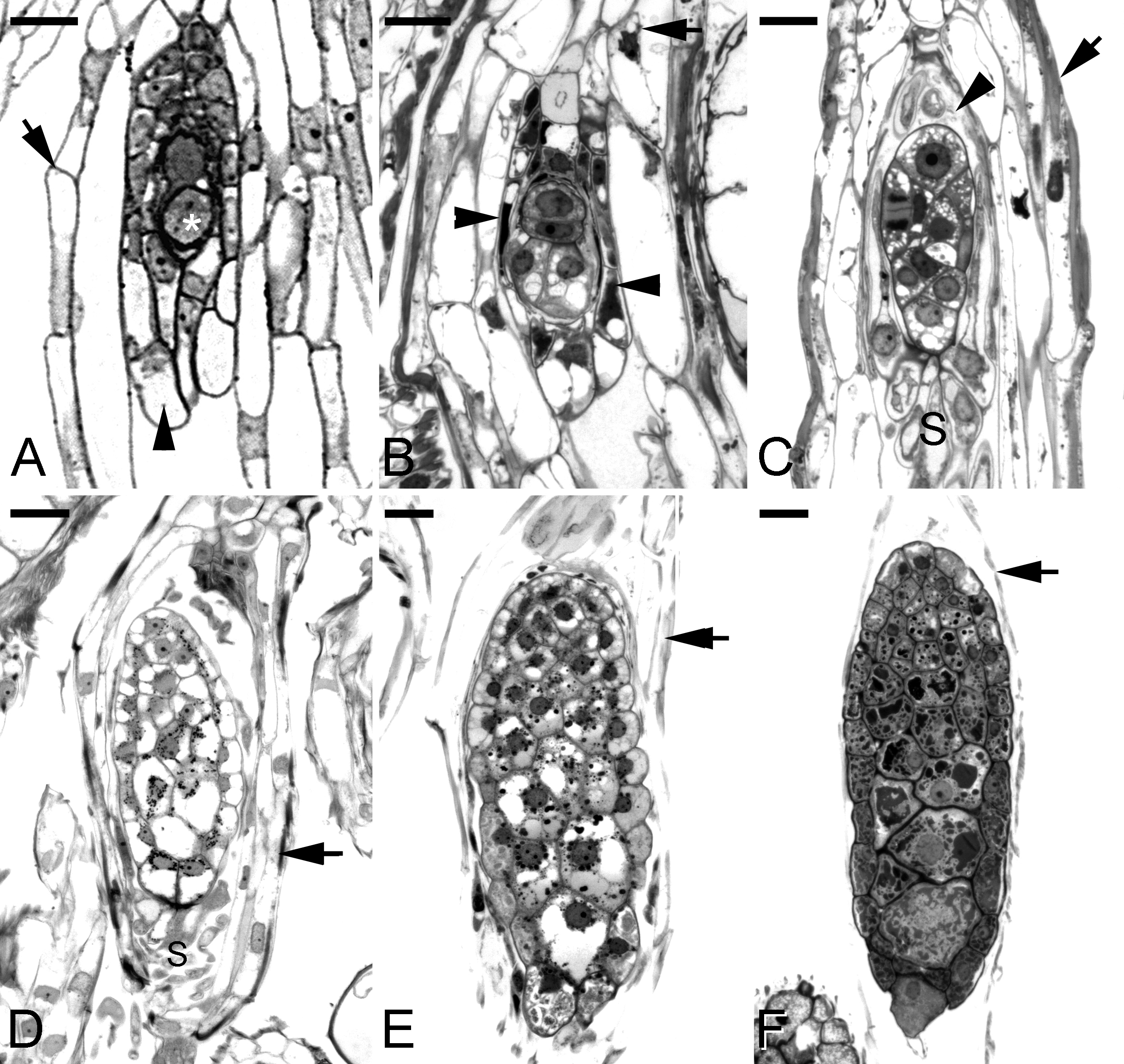

Supplement: Supplementary file 3 — Additional file 3. The seed coat development of Phalaenopsis aphrodite. A After fertilization, the zygote (*) has a dense cytoplasm, and the inner seed coat (arrowhead) encloses the embryo sac completely. The outer seed coat (arrow) cells have expanded and elongated by vacuolation. Scale bar = 20 μm. B At the four-celled embryo stage, the two cells toward the chalazal end are small with dense cytoplasm, while the other two cells toward the micropylar end continue to enlarge. The cells of the inner seed coat (arrowheads) begin to condense and then degenerate. The cells of the outer seed coat (arrow) also begin to condense, but they still stay turgid. Scale bar = 20 μm. C At the early globular stage, the suspensor cells (S) have elongated and surround the developing embryo proper. The cells of the inner seed coat (arrow) have degenerated completely, and the radial walls of the outer seed coat (arrowhead) have become thickened. Scale bar = 20 μm. D At the globular stage, a cell size gradient is noted in the embryo proper, with smaller cells in the chalazal region and larger cells toward the micropylar end. The suspensor cells (S) and the cells of the outer seed coat (arrow) are undergoing dehydration and becoming shriveled. Scale bar = 20 μm. E As development progresses, the embryo cells become cytoplasmic with dense cytoplasm. The seed coat has shriveled (arrow). Scale bar = 20 μm. F At maturity, the embryo cells have an abundant reserves deposit. The embryo is enveloped by the shriveled seed coat (arrow). Scale bar = 20 μm [file 40529_2023_400_MOESM3_ESM.jpg]
